# Targeting Dengue Virus NS-3 Helicase by Ligand based Pharmacophore Modeling and Structure based Virtual Screening

**DOI:** 10.3389/fchem.2017.00088

**Published:** 2017-10-31

**Authors:** Sobia A. Halim, Shanza Khan, Ajmal Khan, Abdul Wadood, Fazal Mabood, Javid Hussain, Ahmed Al-Harrasi

**Affiliations:** ^1^Department of Biochemistry, Kinnaird College for Women, Lahore, Pakistan; ^2^Department of Chemistry, COMSATS Institute of Information Technology, Abbottabad, Pakistan; ^3^UoN Chair of Oman Medicinal Plants and Marine Products, University of Nizwa, Nizwa, Oman; ^4^Department of Biochemistry, Shankar Campus, Abdul Wali Khan University Mardan, Mardan, Pakistan; ^5^Department of Biological Sciences and Chemistry, College of Arts and Sciences, University of Nizwa, Nizwa, Oman

**Keywords:** dengue virus non-structural protein-3 Helicase, virtual screening, pharmacophore modeling, molecular docking, ADMET, FRED

## Abstract

Dengue fever is an emerging public health concern, with several million viral infections occur annually, for which no effective therapy currently exist. Non-structural protein 3 (NS-3) Helicase encoded by the dengue virus (DENV) is considered as a potential drug target to design new and effective drugs against dengue. Helicase is involved in unwinding of dengue RNA. This study was conducted to design new NS-3 Helicase inhibitor by *in silico* ligand- and structure based approaches. Initially ligand-based pharmacophore model was generated that was used to screen a set of 1201474 compounds collected from ZINC Database. The compounds matched with the pharmacophore model were docked into the active site of NS-3 helicase. Based on docking scores and binding interactions, 25 compounds are suggested to be potential inhibitors of NS3 Helicase. The pharmacokinetic properties of these hits were predicted. The selected hits revealed acceptable ADMET properties. This study identified potential inhibitors of NS-3 Helicase *in silico*, and can be helpful in the treatment of Dengue.

## Introduction

Dengue is one of the most common infections in tropical and subtropical countries, and one of the major diseases in Pakistan since 2005. Over past few years around 48,910 cases appeared with 566 death cases. The first deadly outbreak was reported in Lahore in 2011, where 21,685 cases with 350 deaths were recorded (Lindenbach and Rice, 2003; Mukhtar et al., 2011; Ali et al., 2013; Khan et al., 2013, 2014, 2015, 2016; Rasheed et al., 2013; Raza et al., 2014), while about 50–100 million cases appeared worldwide. Dengue is caused by dengue virus (DENV) which belongs to the Flavivirus genus of the Flaviviridae family (Lindenbach and Rice, 2003). The infected dengue cases show several clinical symptoms including high fever, headache, muscular pain, and nausea/vomiting which can lead to serious conditions such as dengue shock syndrome (DSS) or dengue hemorrhagic fever (DHF). DSS/DHF can eventually cause death within 24 h. Till date no effective drugs are available to cure the disease completely (Kuhn et al., 2002; van Gorp et al., 2002; Seneviratne et al., 2006; Bhatt et al., 2013). Therefore, new and effective inhibitors are needed to be designed as potential therapeutics to cure the disease.

DENV consists of positive stranded RNA composed of a 5′ untranslated region (UTR), a large open reading frame, and a 3′ UTR (Lindenbach and Rice, 2003). The viral genome is translated into three structural proteins: C (Capsid), prM (Membrane precursor), E (Envelop) and seven non-structural proteins (NS1-NS2A-NS2B-NS3-NS4A-NS4B-NS5) (Kuhn et al., 2002).

Viral particles appear as smooth surfaces of DENV-E dimers arranged in a head-to-tail fashion parallel to the viral membrane (Modis et al., 2003). The crystal structure of E revealed each monomer consists of three domains that plays different roles in the virus life cycle (Gubler and Clark, 1995; Modis et al., 2005). E is involved in receptor binding, induction of antibody responses, viral fusion and assembly (Gubler and Clark, 1995). The other viral proteins help in RNA packaging and assembly. Capsid protein encapsidates viral RNA and interacts with the genetic material. Membrane glycoprotein is the mature form of pre-membrane (prM) which acts as a chaperone for E. During viral egress through the trans-Golgi network, host furin protein cleaves prM to M. Following cleavage, viral particles are considered mature (Lindenbach and Rice, 2003). The seven DENV non-structural proteins are essential in the virus life cycle. NS1 play role in signaling and replication of virus RNA. NS2A is essential for viral replication and packaging. NS2B is serine protease which acts as a co-factor for NS3. NS3 is a multifunctional protein that posses serine protease, helicase (DENV NS3H), RNA-stimulated nucleoside tri-phosphatase (NTPase/ATPase/helicase), and RNA 5′-triphosphatase (RTPase) activities which are essential for viral RNA replication and capping (Kadaré and Haenni, 1997; Singleton and Wigley, 2002; Wang et al., 2009). These proteins have 67% similarity in four strains of DENV (DENV1-4). These enzymes are important in replication and translation process. NS4A and NS4B are transmembrane proteins responsible for the membrane arrangements leading to the formation of the viral replication complex (Nemésio et al., 2012). Along with NS2A, these proteins have been implicated in interferon antagonism (Muñoz-Jordán et al., 2005). NS5 protein is a RNA-dependent RNA polymerase (Lindenbach and Rice, 2003).

Currently DENV enzymes are targeted to design anti-viral therapies. In this study NS3 helicase was targeted. The helicase domain resides within the region of 170–618 residues of NS3 protein. NS3 helicase unwinds double stranded RNA to release single stranded RNA, which is then used as a template for NS5 protein in replication (Bartelma and Padmanabhan, 2002). Studies showed that the ATPase/helicase and RTPase activities of DENV NS3 share a common active site (Borowski et al., 2001; Benarroch et al., 2004). However, later on, X-ray crystallographic studies proved that ATPase and helicase activities are conferred by distinct sites (Luo et al., 2008). Functional activities of helicase are well-characterized for other species of the flaviviridae family such as Hepatitis C Virus (HCV), and Yellow fever virus (YFV) etc. (Warrener et al., 1993; Utama et al., 2000). DENV has four antigenically distinct serotypes, DENV 1–4. DENV-2 was prominent in outbreaks in 2011. Phylogenetic analysis of partial DENV-2 sequences has revealed that genotype IV or cosmopolitan genotype of DENV-2 is circulating in Pakistan (Fatima et al., 2011). NS3 and NS5 are conserved within the four serotypes (Li et al., 2005), that permit the design of drugs which could be effective against all dengue virus serotypes and other related flaviviruses (Xu et al., 2005; Keller et al., 2006). The structural details of DENV helicase in complex with ssRNA (Luo et al., 2008) and in apo form (Xu et al., 2005) are available at good resolution which opens the opportunities to design novel drugs against dengue.

Computational tools have a large impact in drug discovery because of its fast and promising results. *In silico* techniques are categorized as structure- and ligand based. Both structure and ligand based methods are used to predict binding affinities of newly designed compounds. With our interest in computational analysis of several biologically important drug targets (Halim et al., 2013, 2015; Halim and Zaheer-ul-Haq, 2015), we conducted this study to identify novel and effective DENV NS3-helicase inhibitors *in silico*. The compound which shows potential will be selected for biological testing in future to accelerate the therapeutic process against dengue.

## Materials and methods

**Scheme 1:**
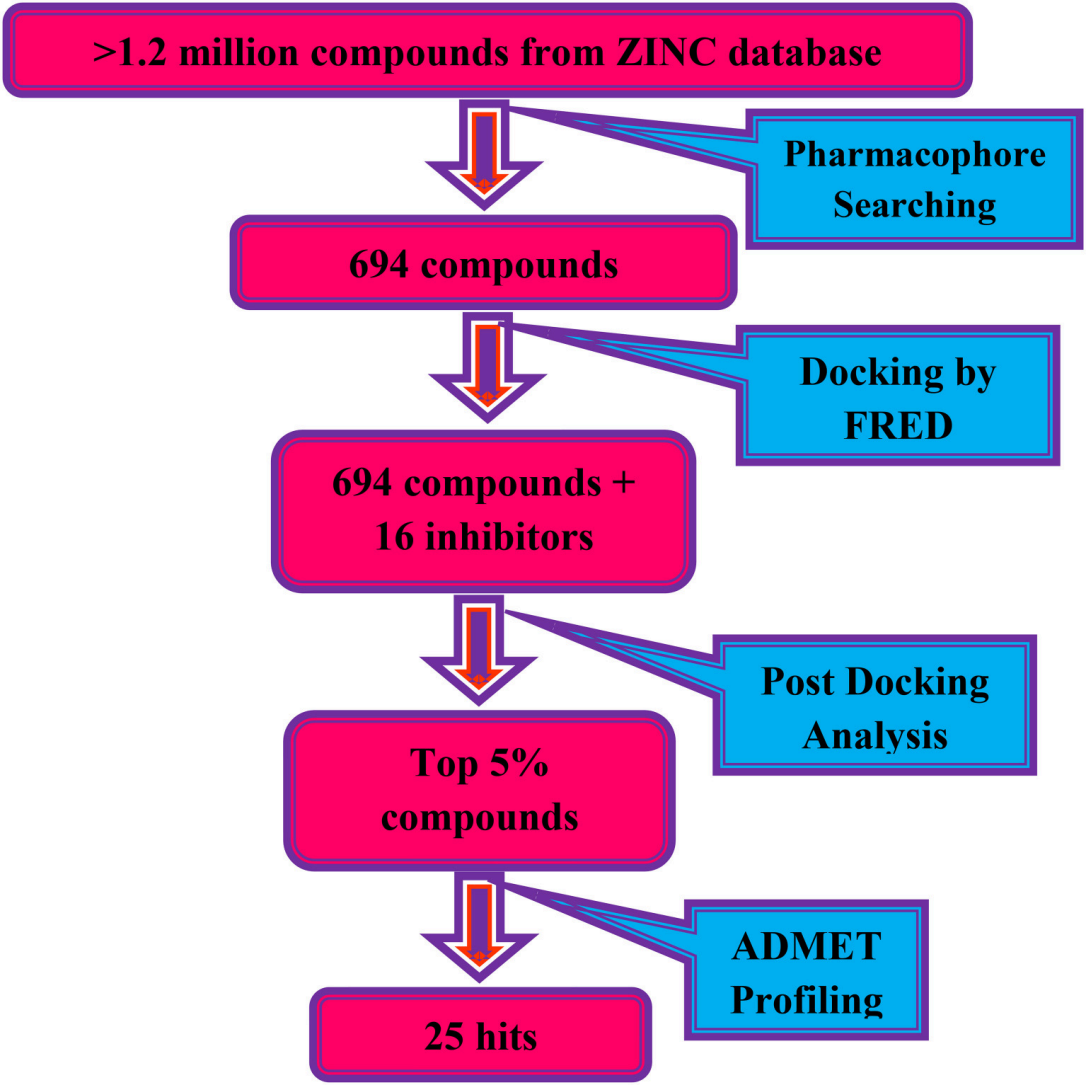
The virtual screening work flow.

All the computation experiments were performed on Windows 8 workstation. LigandScout version 3.12 (Wolber and Langer, 2005) was used for pharmacophore modeling. The X-ray crystallography structure of DENV-2 NS-3 helicase [PDB ID: 2BMF (Xu et al., 2005)] was retrieved from RCSB Protein Data Bank (https://www.rcsb.org) (Berman et al., 2000) for molecular docking. Docking was performed on FRED (Fast Rigid Exhaustive Docking) (McGann, 2012). Protein-ligand interactions were visualized on Chimera software (Pettersen et al., 2004). ADMET properties were calculated on admetSAR server (Cheng et al., 2012). The flow of work is shown in Scheme 1.

### Pharmacophore modeling and screening of ZINC database

The pharmacophore model was generated by LigandScout (Wolber and Langer, 2005) using three most active inhibitors of DENV NS-3 Helicase (Table 1). The known inhibitors were selected from literature (Mastrangelo et al., 2012; Basavannacharya and Vasudevan, 2014; Sweeney et al., 2015). The compounds structures and IC_50_ values are shown in Table 1. **Lig and Scout** generates pharmacophore model by using structural data from protein-ligand complex structures or from small compounds. Subsequently protein-ligand interactions are depicted by chemical features including H-bond donors, H-bond acceptors, lipophilic areas, positively and negatively ionizable chemical groups. A pattern-matching based alignment method is used to align the generated pharmacophores. The aligned pharmacophore from different complexes are used to either create “shared feature pharmacophore (SFP)” or “merged feature pharmacophore (MFP).” SFP shares common interactions of several complexes, while MSP comprises of extended pharmacophore. We used SFP method. 1201474 compounds from “Drug Now” category of ZINC database (Irwin et al., 2012) was selected as a screening library. The pharmacophore was validated by adding the known inhibitors of DENV NS-3 Helicase in the screening database. The compounds that matched with pharmacophore model were docked into the protein by FRED docking program.

**Table 1:** The Chemical structures and IC_50_ values of 16 known inhibitors of NS-3 Helicase.

**Compound ID**	**Compounds chemical structure**	**IC_50_ Values/μM**	**Reference**
1	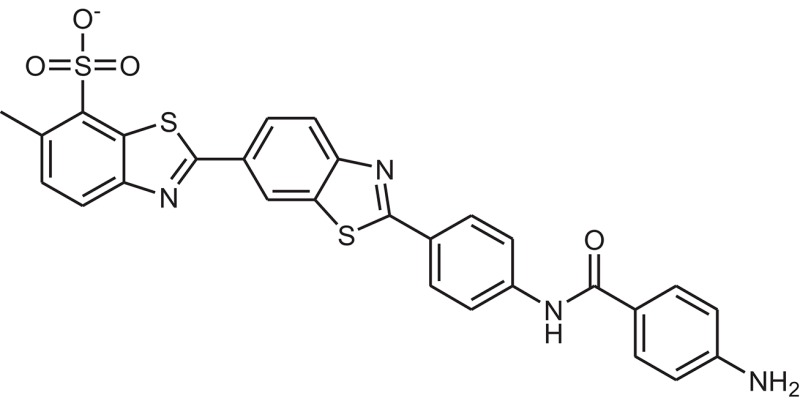	7.6 ± 1.6	Sweeney et al., 2015
2	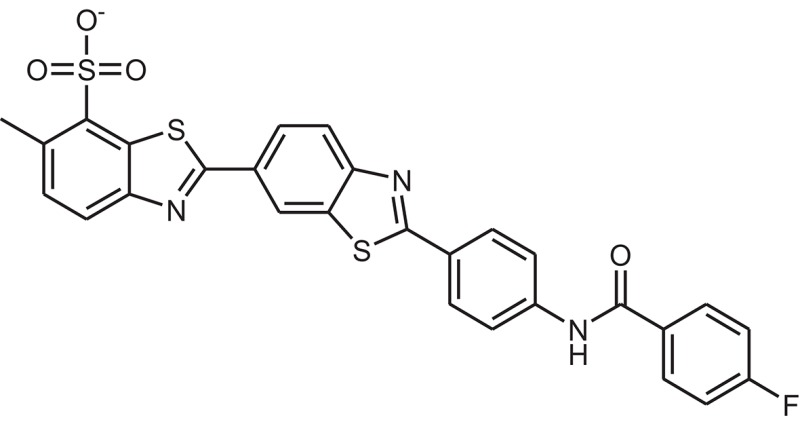	6.6 ± 0.9	Sweeney et al., 2015
3	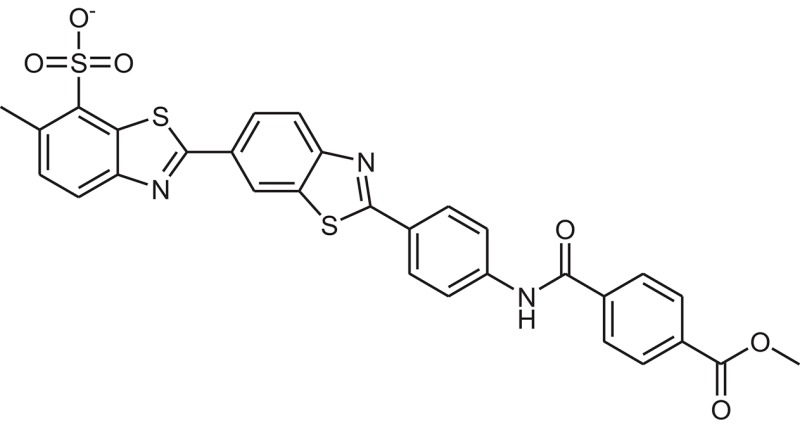	7.9 ± 2.5	Sweeney et al., 2015
4	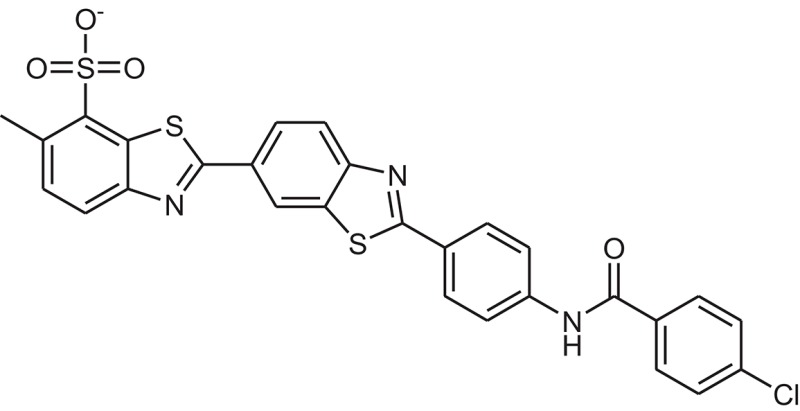	5.6 ± 1.2	Sweeney et al., 2015
5	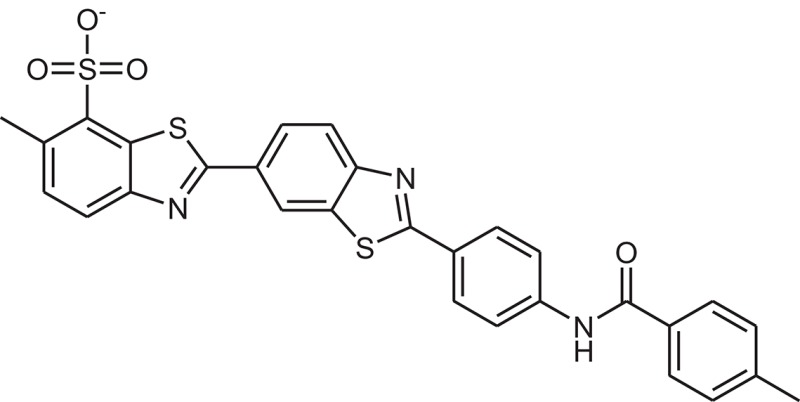	4.3 ± 1.9	Sweeney et al., 2015
6	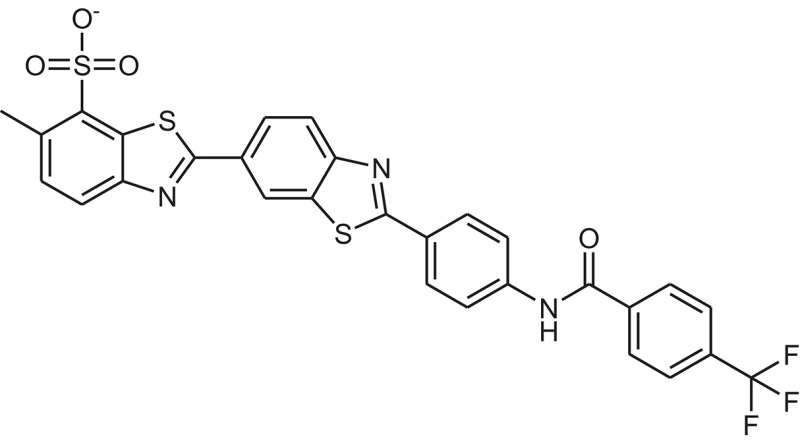	5.2 ± 0.6	Sweeney et al., 2015
7	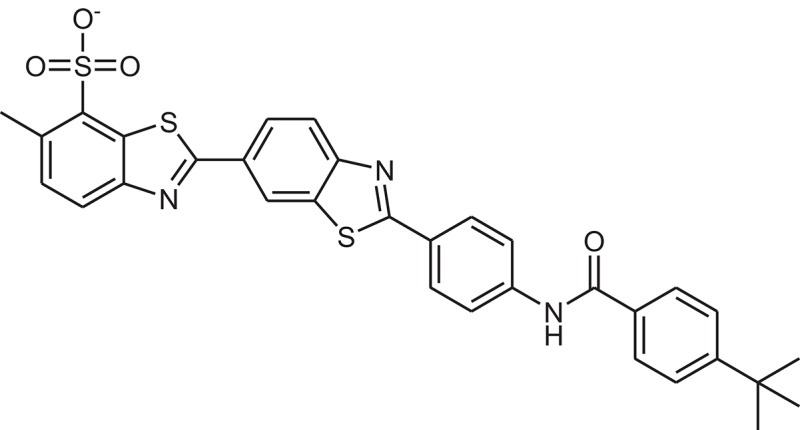	10 ± 3.4	Sweeney et al., 2015
8	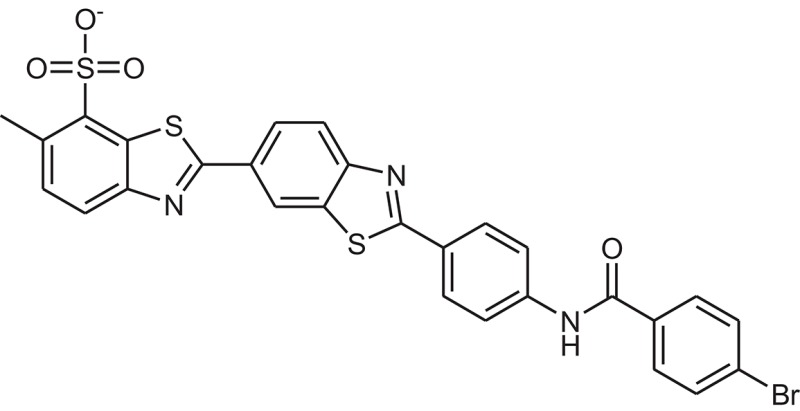	5.0 ± 1.2	Sweeney et al., 2015
9	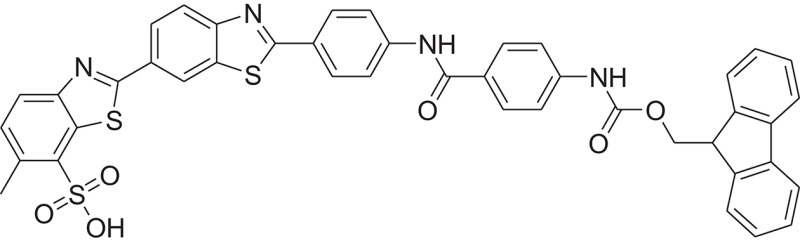	3.5 ± 1.7	Sweeney et al., 2015
10	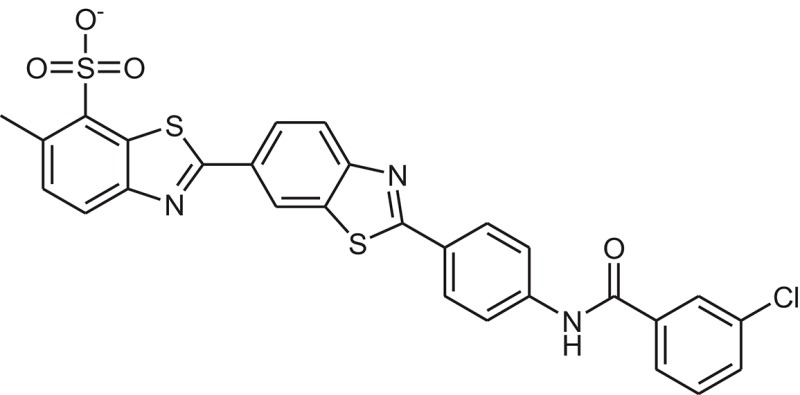	5.1 ± 0.9	Sweeney et al., 2015
11	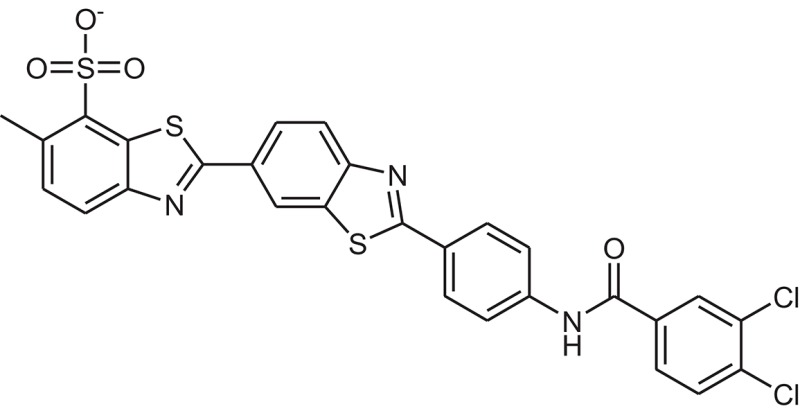	3.6 ± 0.9	Sweeney et al., 2015
12	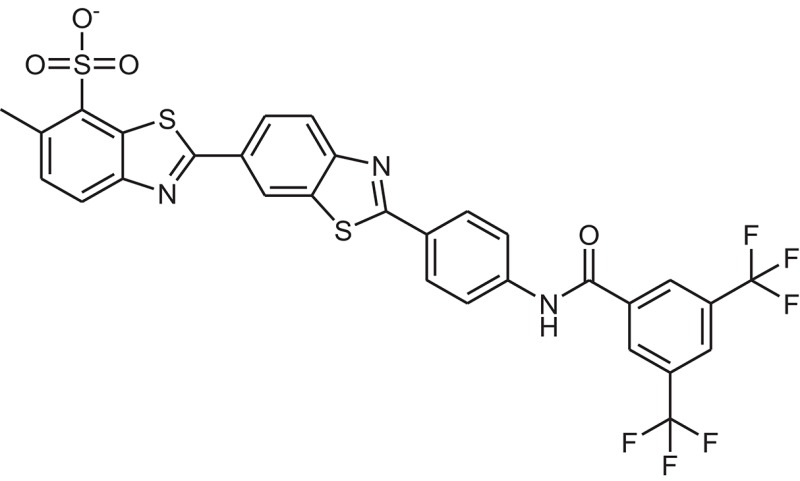	7.7 ± 1.8	Sweeney et al., 2015
13	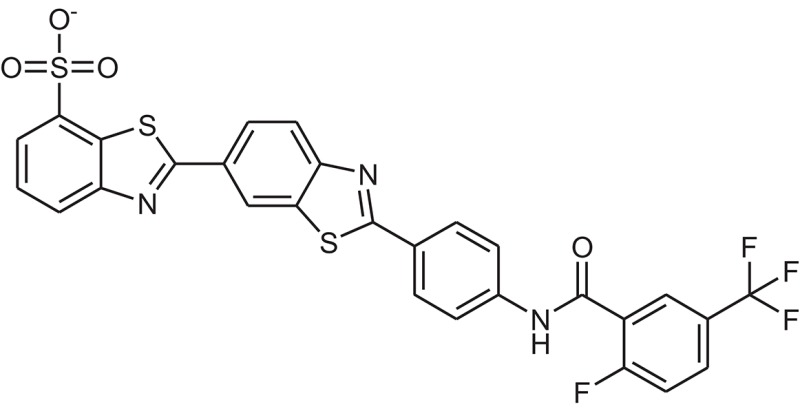	5.4 ± 1.3	Sweeney et al., 2015
14	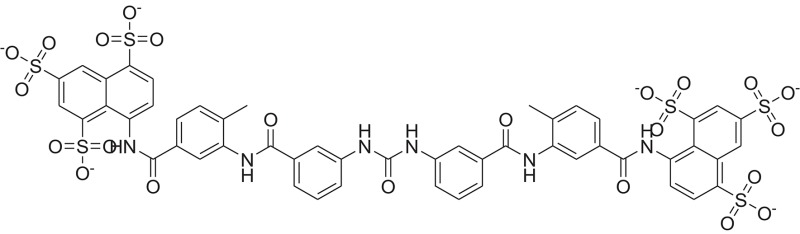	0.4	Basavannacharya and Vasudevan, 2014
15	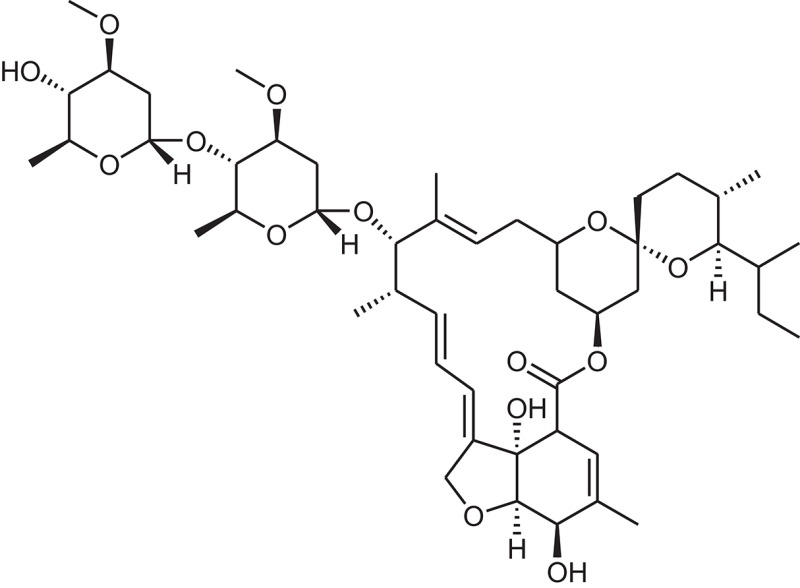	0.5 ± 0.07	Mastrangelo et al., 2012
16	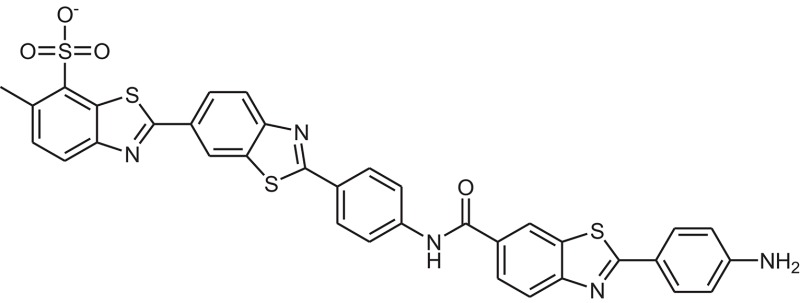	1.5 ± 0.2	Mastrangelo et al., 2012

### Fred docking protocol

FRED performs rigid docking using exhaustive search algorithm (McGann, 2012) that requires pre-made multiconformer library of ligand. Exhaustive search algorithm rotates and translates each conformation of compound in the protein's binding site to select the best pose that do not clash with the active site or extend far away. Subsequently the best poses are assigned a score. Initially ligand conformations were generated by Omega 2.4.6 (Hawkins et al., 2010). The maximum number of conformations was set as 10 for each ligand along with a dielectric constant of 1.0 and the search force field mmff94s_NoEstat. The 3D structure of DENV NS-3 Helicase (PDB code: 2BMF, resolution: 2.4Å) was retrieved from PDB. The protein file was prepared on FRED make receptor 2.2.5 software. Missing atoms and bonds of protein was checked. Docking box was constructed on the single stranded RNA binding site with a volume of 18869Å^3^. Inner and outer counter was set as 12Å^3^ and 6673Å^3^, respectively. Eight scoring functions (McGann, 2012): Shapegauss (SG), Piecewise Linear Potential (PLP), ChemScore, Chemgauss 2 (CG2), and Chemgauss 3 (CG3), OeChemSscore, ScreenSscore and Zapbind were used in docking. The receptor file was docked with multiple conformer libraries of ligands and top fifty poses of each ligand were saved for further analysis.

### Admet prediction

ADMET properties predict the absorption, distribution, metabolism, excretion, and toxicity of compounds in and through the human body. It estimates pharmacokinetic and pharmacodynamic profiles of drugs, and plays crucial role in drug development. The ADMET properties of the selected ligands were estimated by online server admetSAR (Cheng et al., 2012). admetSAR collects data of diverse compounds associated with ADMET properties from literature, and provides ADMET structure-activity relationship models to predict ADMET properties of drug candidates.

## Results

### Pharmacophore modeling and virtual screening

**Figure 1:**
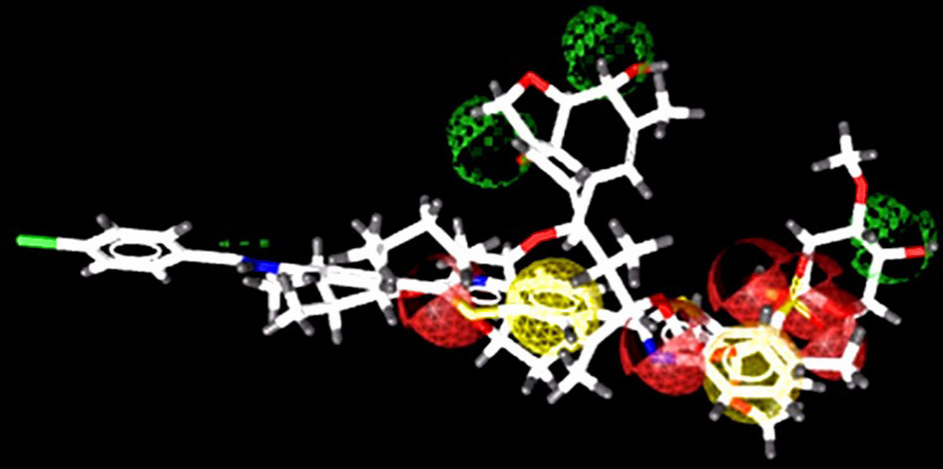
Pharmacophore model consist of 5 H-Bond acceptors (Red spheres), 4 H-Bond donors (Green spheres), and 2 hydrophobic features (Yellow spheres). The known inhibitors are depicted in stick models.

ZINC provides chemical molecules repositories that contain millions of diverse compounds. >1.2 million compounds were retrieved from the “Drug Now” category of ZINC database. To reduce the size of the dataset for docking, ligand-based pharmacophore model was constructed *via* LigandScout based on 3D structure of three most active known inhibitors (Compound ID: **10**, **14**, and **15**). The pharmacophore model was composed of 5 Hydrogen Bond Acceptors (Red spheres), 4 Hydrogen Bond Donors (Green spheres) and 2 hydrophobic features (Yellow spheres) (Figure 1). 694 compounds were matched with the pharmacophore query and 16 known inhibitors were subjected to molecular docking.

### Molecular docking

The compounds retrieved by pharmacophore based screening were docked into the NS-3 Helicase active site by FRED. After docking, the results of the eight scoring functions were compared. Those scoring functions were selected that ranked all 16 known inhibitors at the top of its ranking list. This retrospective analysis shows that Chemgauss2 (CG2) and Shapegauss (SG) placed all the known inhibitors at the top of their docking results (Table 2). Subsequently consensus strategy was used for the selection of best predicted hits. Based on CG2 and SG ranking, top 5% compounds were selected as hits. The binding interactions analysis of the selected hits showed that 25 compounds acts as potential NS-3 inhibitors. The chemical structures and ZINC codes of selected 25 hits are shown in Table 3, while docking results are tabulated in Table 4.

**Table 2:** FRED docking results of Known inhibitors (scores and rank).

	**FRED docking results**			
**Compound ID**	**CG2 Score**	**Rank**	**SG Score**	**Rank**
1	−116.18	1	−917.35	2
2	−103.52	2	−808.91	3
3	−100.03	3	−1, 019.39	1
4	−82.02	4	−764.97	4
5	−76.16	5	−667.81	8
6	−71.74	6	−697.54	5
7	−71.22	7	−667.42	9
8	−69.92	9	−691.82	6
9	−69.10	10	−649.18	10
10	−68.72	11	−628.09	15
11	−68.36	12	−635.27	12
12	−67.02	15	−631.03	14
13	−66.89	17	−674.01	7
14	−66.68	19	−608.07	17
15	−66.06	20	−609.41	16
16	−66.06	21	−642.12	11

*CG2, Chemgauss 2; SG, Shapegauss*.

**Table 3:** The chemical structures of 25 hits selected after post docking analysis.

**Compound ID**	**ZINC ID**	**Chemical structure**	**Chemical name**
Z1	ZINC06810538	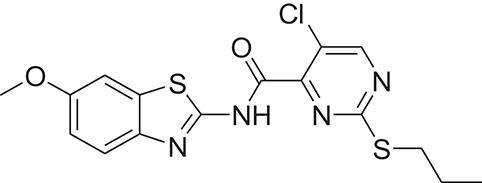	5-chloro-N-(6-methoxybenzothiazol-2-yl)-2-propylsulfanyl-pyrimidine-4-carboxamide
Z2	ZINC22690487	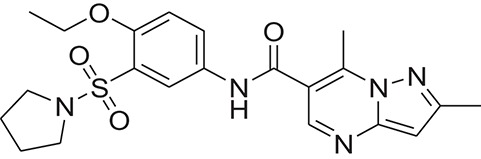	N-(4-ethoxy-3-pyrrolidin-1-ylsulfonyl-phenyl)-2,7-dimethyl-pyrazolo[1,5-a]pyrimidine-6-carboxamide
Z3	ZINC13006693	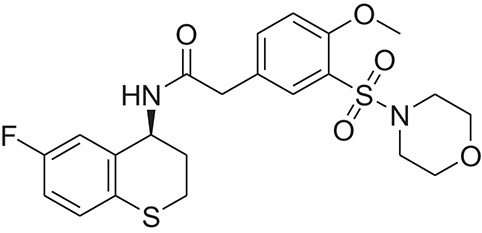	N-[(4S)-6-fluorothiochroman-4-yl]-2-(4-methoxy-3-morpholinosulfonyl-phenyl)acetamide
Z4	ZINC16682175	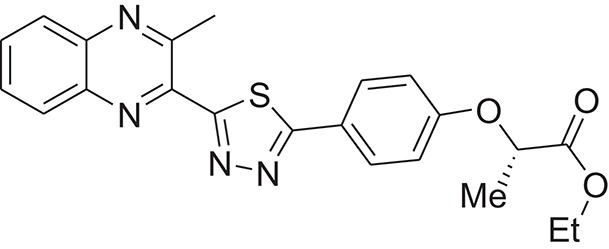	(2S)-ethyl 2-(4-(5-(3-methylquinoxalin-2-yl)-1,3,4-thiadiazol-2-yl)phenoxy)propanoate
Z5	ZINC27794792	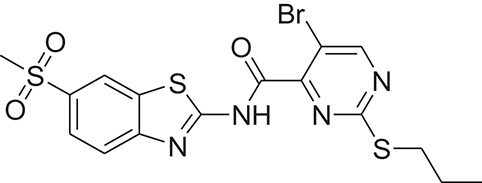	5-bromo-N-(6-methylsulfonyl-1,3-benzothiazol-2-yl)-2-propylsulfanyl-pyrimidine-4-carboxamide
Z6	ZINC00915026	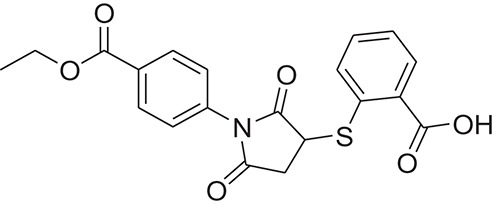	2-[1-(4-ethoxycarbonylphenyl)-2,5-dioxo-pyrrolidin-3-yl]sulfanylbenzoic
Z7	ZINC29761473	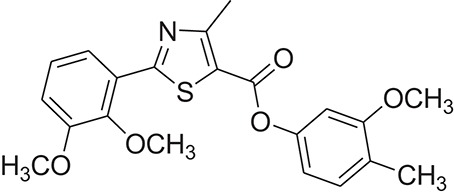	3-methoxy-4-methylphenyl 2-(2,3-dimethoxyphenyl)-4-methylthiazole-5-carboxylate
Z8	ZINC98086540	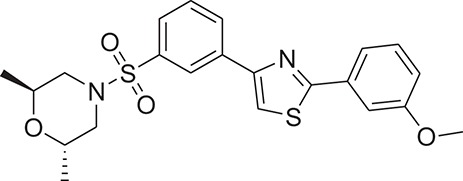	(2S,6S)-4-[3-[2-(3-methoxyphenyl)thiazol-4-yl]phenyl]sulfonyl-2,6-dimethyl-morpholine
Z9	ZINC35476132	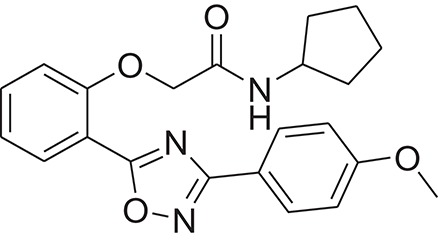	N-cyclopentyl-2-(2-(3-(4-methoxyphenyl)-1,2,4-oxadiazol-5-yl)phenoxy)acetamide
Z10	ZINC14662615	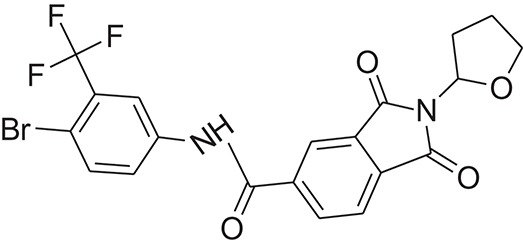	N-[4-bromo-3-(trifluoromethyl)phenyl]-1,3-dioxo-2-[tetrahydrofuran-2-yl]isoindoline-5-carboxamide
Z11	ZINC11852541	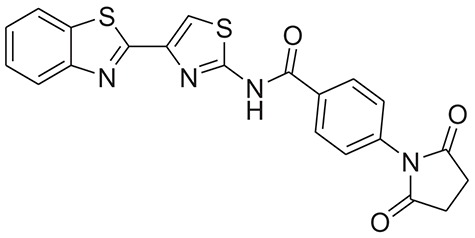	N-[4-(1,3-benzothiazol-2-yl)thiazol-2-yl]-4-(2,5-dioxopyrrolidin-1-yl)benzamide
Z12	ZINC29389407	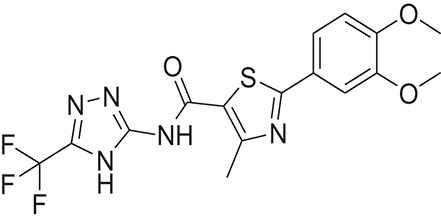	2-(3,4-dimethoxyphenyl)-4-methyl-N-[5-(trifluoromethyl)-4H-1,2,4-triazol-3-yl]thiazole-5-carboxamide
Z13	ZINC16045701	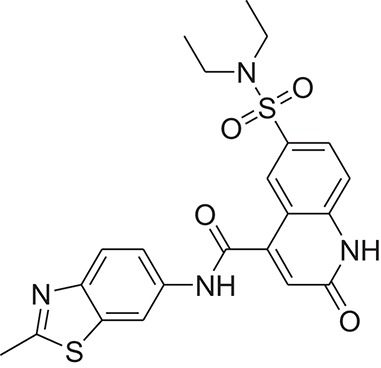	6-(diethylsulfamoyl)-2-hydroxy-N-(2-methyl-1,3-benzothiazol-6-yl)quinoline-4-carboxamide
Z14	ZINC27742665	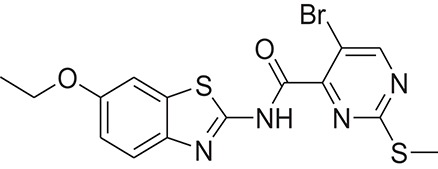	5-bromo-N-(6-ethoxy-1,3-benzothiazol-2-yl)-2-methylsulfanyl-pyrimidine-4-carboxamide
Z15	ZINC35553300	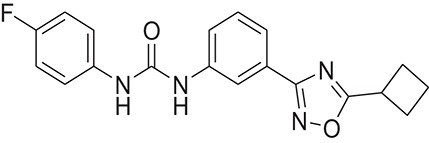	1-[3-(5-cyclobutyl-1,2,4-oxadiazol-3-yl)phenyl]-3-(4-fluorophenyl)urea
Z16	ZINC01106038	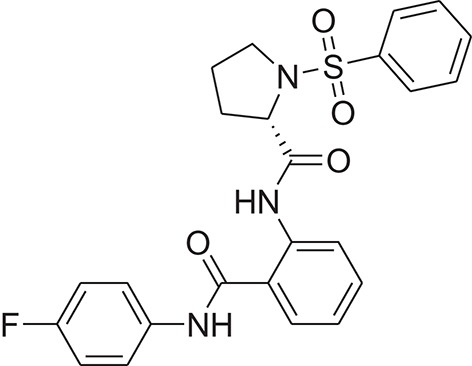	(2S)-1-besyl-N-[2-[(4-fluorophenyl)carbamoyl]phenyl]pyrrolidine-2-carboxamide
Z17	ZINC10220409	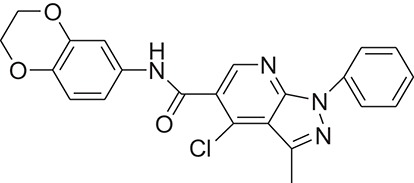	4-chloro-N-(2,3-dihydrobenzo[b][1,4]dioxin-6-yl)-3-methyl-1-phenyl-1H-pyrazolo[3,4-b]pyridine-5-carboxamide
Z18	ZINC00878954	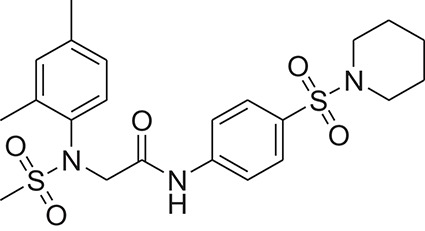	2-(N-mesyl-2,4-dimethyl-anilino)-N-(4-piperidinosulfonylphenyl)acetamide
Z19	ZINC35481917	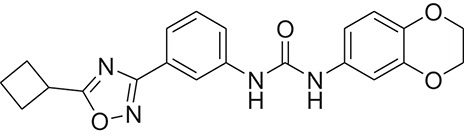	1-[3-(5-cyclobutyl-1,2,4-oxadiazol-3-yl)phenyl]-3-(2,3-dihydro-1,4-benzodioxin-6-yl)urea
Z20	ZINC07916471	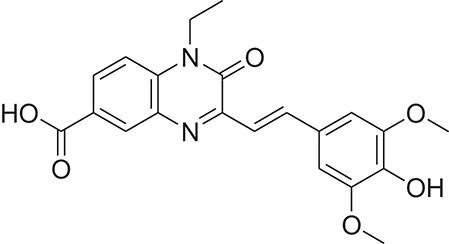	(E)-3-(4-hydroxy-3,5-dimethoxystyryl)-1-ethyl-2-oxo-1,2-dihydroquinoxaline-6-carboxylic acid
Z21	ZINC39124407	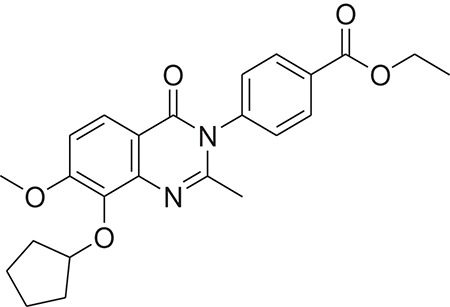	Benzoic acid, 4-[8-(cyclopentyloxy)-7-methoxy-2-methyl-4-oxo-3(4H)-quinazolinyl]-, ethyl ester
Z22	ZINC20896686	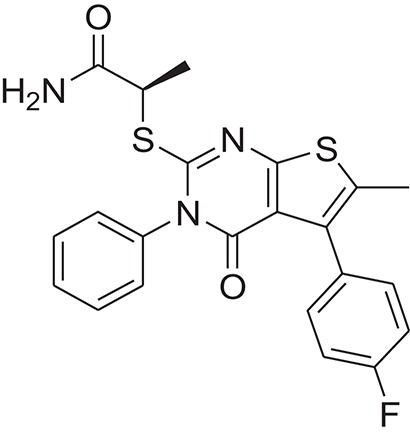	(2R)-2-[5-(4-fluorophenyl)-6-methyl-4-oxo-3-phenyl-thieno[2,3-d]pyrimidin-2-yl]sulfanylpropanamide
Z23	ZINC07574924	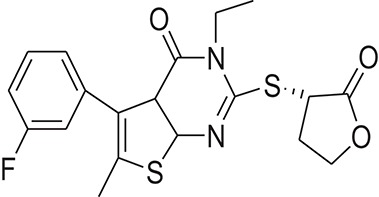	3-ethyl-5-(3-fluorophenyl)-6-methyl-2-((S)-2-oxo-tetrahydrofuran-3-ylthio)thieno[2,3-d]pyrimidin-4(3H,4aH,7aH)-one
Z24	ZINC65153953	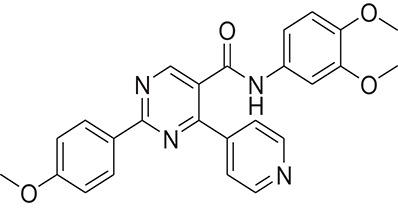	N-(3,4-dimethoxyphenyl)-2-(4-methoxyphenyl)-4-(4-pyridyl)pyrimidine-5-carboxamide
Z25	ZINC65153979	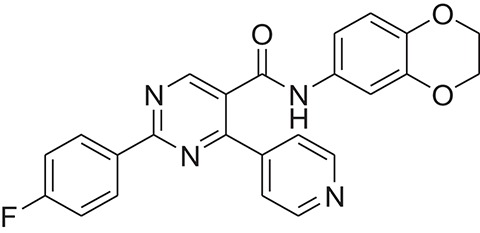	N-(2,3-dihydro-1,4-benzodioxin-6-yl)-2-(4-fluorophenyl)-4-(4-pyridyl)pyrimidine-5-carboxamide

**Table 4:** FRED Docking Results of the 25 best selected Compounds.

**Comp. ID**	**CG2**	**SG**	**PLP**	**CG3**	**CS**	**OECS**	**SS**	**Zapbind**
Z1	−67.21	−514.22	−67.00	−75.11	−23.07	−40.88	−145.23	−23.11
Z2	−66.96	−533.63	−49.55	−64.70	−11.22	−37.74	−142.46	−25.04
Z3	−65.52	−543.83	−60.88	−66.88	−11.73	−32.12	−142.24	−18.42
Z4	−64.88	−572.75	−63.38	−71.98	−19.31	−40.33	−151.69	−29.05
Z5	−64.81	−525.27	−55.03	−63.33	−16.52	−28.08	−151.22	−21.42
Z6	−63.82	−491.53	−56.93	−82.35	−16.56	−36.44	−129.97	−25.52
Z7	−62.67	−494.47	−58.38	−63.00	−16.60	−36.61	−142.99	−30.19
Z8	−62.28	−568.90	−52.44	−69.81	−13.81	−33.15	−142.58	−16.72
Z9	−62.03	−502.51	−56.80	−75.71	−19.40	−40.22	−137.54	−26.82
Z10	−61.00	−491.13	−60.10	−71.13	−16.73	−33.80	−159.33	−19.08
Z11	−60.59	−526.49	−68.01	−90.00	−17.27	−39.41	−139.96	−25.15
Z12	−58.10	−525.62	−54.29	−64.31	−10.27	−40.53	−131.50	−9.33
Z13	−60.20	−514.26	−61.60	−59.55	−17.52	−38.12	−158.27	−18.85
Z14	−60.17	−492.93	−62.44	−64.82	−21.92	−36.76	−129.57	−14.16
Z15	−59.21	−492.27	−68.92	−72.79	−19.21	−37.51	−172.01	−22.96
Z16	−59.06	−527.16	−46.31	−48.49	−9.53	−32.04	−131.45	−14.68
Z17	−58.99	−497.64	−56.71	−73.47	−20.56	−35.15	−158.51	−9.913
Z18	−58.97	−534.68	−44.37	−51.75	−4.89	−26.83	−114.48	−21.13
Z19	−58.31	−495.88	−59.00	−74.32	−19.43	−37.81	−124.93	−30.57
Z20	−58.26	−533.37	−57.62	−74.36	−15.45	−39.39	−165.67	9.68
Z21	−60.21	−520.61	−52.45	−74.25	−19.97	−36.61	−142.99	−30.19
Z22	−57.73	−503.58	−51.21	−69.29	−20.54	−36.85	−127.14	4.96
Z23	−57.58	−494.40	−52.34	−59.86	−17.72	−35.64	−122.66	19.48
Z24	−56.70	−503.03	−43.80	−69.87	−17.79	−34.81	−98.66	−30.22
Z25	−56.33	−503.89	−54.82	−78.28	−17.81	−34.79	−141.48	−21.99

*CG2, Chemgauss 2; CG3, Chemgauss 3; SG, Shapegauss; PLP, Piecewise Linear Potential; CS, ChemScore; OECS, OEChemscore; SS, ScreenScore*.

### Binding interaction analysis

**Figure 2:**
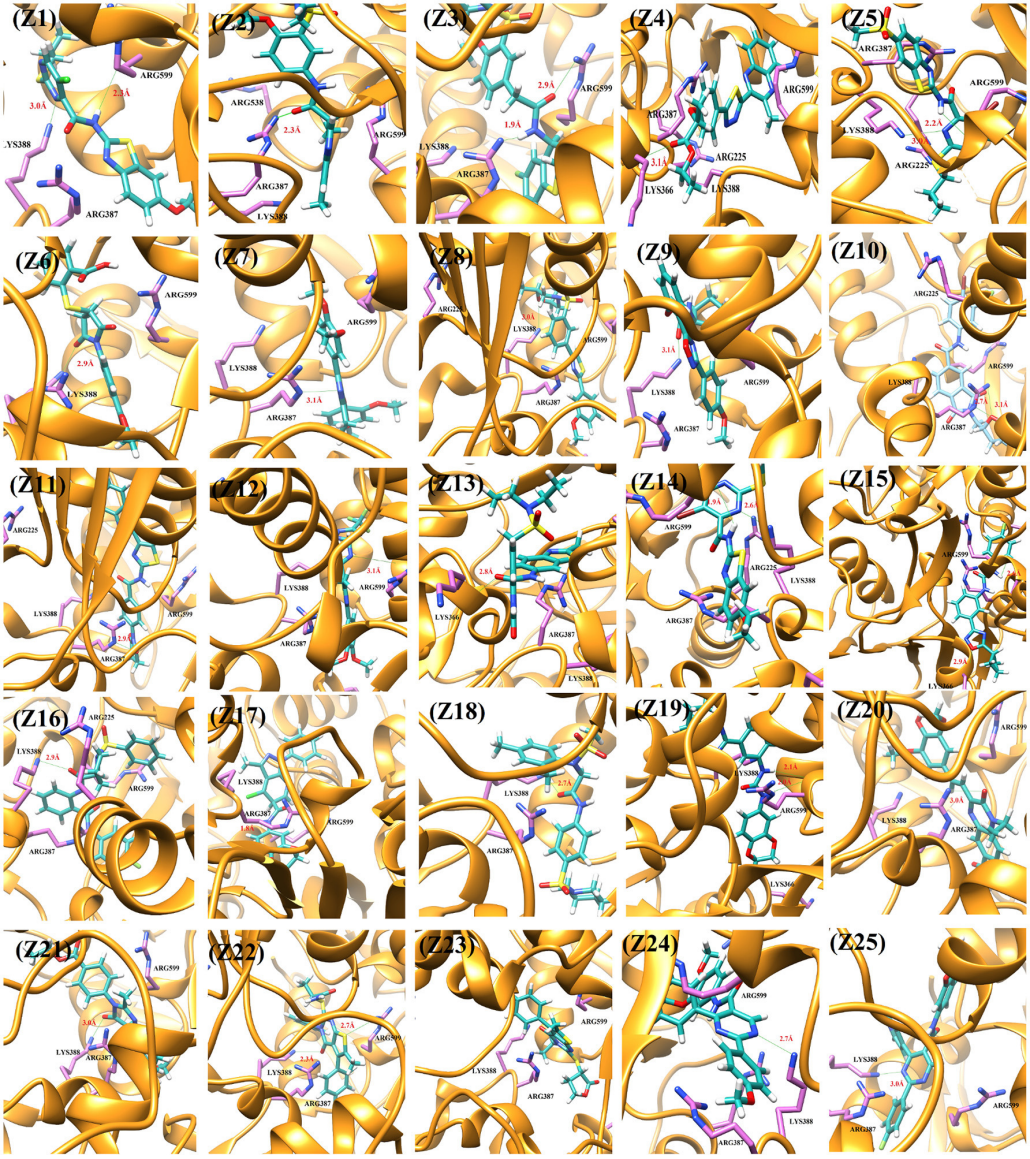
The DENV NS-3 Helicase structure is depicted in Golden ribbon. The residues of active site are depicted as purple sticks. The predicted inhibitors are displayed as blue stick model.

The docked view of the selected compounds are depicted in Figure 2. The binding mode of the compound **Z1** showed that the compound forms H-bond with Lys388 and Arg599. The calculated H-bond distance between pyrimidine nitrogen of **Z1** and side chain of Lys388 is 3.0Å, while pyrimidine moiety and side chain of Arg599 is 2.3Å. Furthermore, the side chains of surrounding residues stabilize the compound in the active site of NS3. The carbonyl oxygen of compound **Z2** formed H-bond with the amino side chain of Arg387 at a distance of 2.3Å. The carbonyl and amino group of compound **Z3** mediates H-bond with the amino side chain of Arg599 (2.9Å) and side chain of Arg387 (1.9Å), respectively. The docked view of the compound **Z4** shows that this compound is mainly stabilized in the binding site *via* hydrophobic interactions, while the carbonyl oxygen mediates weak H-bonding with amino side chain of Lys366 (3.1Å). The bromo-pyrimidine nitrogen of compound **Z5** is H-bonded to amino side chain of Lys388 (3.0Å). The amino group also formed H-bond with the carbonyl group of Arg599 (2.2Å). The compound **Z6** mediates H-bond with Lys388. The pyrrolidine-dione ring accepts H-bonds from amino side chains of Lys388 (2.9Å). The compound **Z7** interacts with Arg599. The phenyl nitrogen formed weak H-bond with the amino side chain of Arg599 (3.1Å). The dimethyl-morpholine moiety of compound **Z8** found oriented toward Lys388 and the oxygen formed H-bond with its amino side chain at a distance of 3.0Å. The other polar groups of the compound do not interact with surrounding residues as the polar moieties of residues are oriented away from the compound. However, Arg387 provides strong hydrophobic interactions to the methoxy benzene moiety of compound **Z8**. The oxygen at the cyclopentyl ring of compound **Z9** interacted with the amino side chain of Lys388 at a distance of 3.1Å. The methoxyphenyl moiety is oriented toward Arg599 showing strong hydrophobic interactions. The tetrahydrofuran ring of compound **Z10** formed bi-dentate interactions with Arg387. The ring is H-bonded to the amino side chain of Arg387 at a distance of 2.7Å and 3.1Å. The compound **Z11** is composed of five rings in which the pyrrolidine ring mediates H-bonding with amino side chain of Arg387 at a distance of 2.9Å. The surrounding residues Arg538, Arg599, Lys399, and Arg387 hydrophobically stabilize the compound. The triazole moiety of compound **Z12** mediates weak H-bond with the carbonyl side chain of Arg599 at a distance of 3.1Å. The quinolone oxygen of compound **Z13** accepts H-bond from the amino group of Lys366. The H-bond distance is 2.8Å. The compound **Z14** is H-bonded to the amino side chain of Lys388 2.6Å and the amino group is H-bonded to Arg599 (1.9Å). Similarly compound **Z15** is stabilized by two H-bonds with Arg599 and Lys366. The oxadiazole ring accepts H-bond from the amino side chain of Lys366 (2.9Å). The amino group attached with the fluoro-benzene ring donates its H-bond to the carbonyl group of Arg599 (2.6Å). The pyrrolidine moiety of compound **Z16** mediates H-bond with the side chain of Lys388 (2.9Å). However, the benzene ring is tilted toward Arg387. The amino nitrogen of compound **Z17** formed H-bond with the carbonyl group of Arg387 with the bond length of 1.8Å. The carbonyl moiety of compound **Z18** accepts H-bond from the amino side chain of Lys388 (2.7Å). The dimethyl substituted benzene ring found oriented toward Arg387. The compound **Z19** mainly interacts with Arg538 and Arg599. The dihydrobenzene substituted amino groups mediates bidentate interactions with carbonyl group of Arg599 with bond length of 2.1Å and 2.0Å. Moreover, compound also interacts with Lys388 *via* hydrophobic interactions. The quinoxalin moiety of compound **Z20** accepts H-bond from the amino side chain of Arg387 (3.0Å). The quinazolin moiety of compound **Z21** mediated H-bond with the amino side chain of Lys388 (3.0Å). The cyclopentane ring of the **Z21** is tilted toward Arg387 thus stabilizing the compound. The pyrimidine moiety of compound **Z22** accepts H-bond from Lys388 and Arg599 at a distance of 2.3Å and 2.7Å, respectively. The fluoro benzene ring is oriented toward Arg387. Compound **Z23** is composed of four rings in which the oxo-tetrahydrofuran ring is tilted toward Arg387. Lys388 and Arg599 provide strong hydrophobic interactions to stabilize the compound. The pyrimidine nitrogen of compound **Z24** is H-bonded to amino side chain of Lys388 (2.7Å). The phenyl ring formed strong hydrophobic interaction with Arg599. The pyrimidine nitrogen of compound **Z25** mediates H-bonding with amino side chain of Lys388 (3.0Å). The binding modes of the selected compounds showed that strong hydrophobic interactions are provided by the surrounding active site residues of the DENV NS-3 Helicase to stabilize these compounds in the active site.

### Admet prediction

ADMET properties were predicted by online admetSAR server. The results are presented in Table 5. The results revealed that all compounds possess good HIA (Human Intestinal Absorption) values. Greater HIA denotes that the compound could be better absorbed from the intestinal tract upon oral administration. Out of 25 compounds, only four compounds showed low HIA. Moreover, all the compounds displayed negative penetration through the Blood-Brain Barrier (BBB), means these compounds do not cross BBB. In terms of metabolism, we found that some compounds inhibit the members of the cytochrome P450 superfamily of enzymes and some are non-inhibitors. A non-inhibitor of CYP450 means that the molecule will not restrict the biotransformation of drugs metabolized by CYP450 enzyme. AMES toxicity test is employed to predict whether a compound is mutagenic or not. All of the compounds are non-mutagenic. Carcinogenic profile also revealed that all the ligands were non-carcinogenic. Acute oral toxicity test showed that the predicted LD_50_ values of these compounds are >500 mg/kg but less that 5,000 mg/kg, suggesting that these compounds do not posses acute oral toxicity at lower doses. Important information obtained from admetSAR server was the computed LD_50_ dose in rat model. Comparing the LD_50_ doses, a compound with lower dose is more lethal than the compound having higher LD_50_. From our observation, it was seen that all compounds possess higher LD_50_ values. The physicochemical properties (Table 5) showed that these compounds acquire drug like properties and can be good inhibitors of DENV NS3 protein when tested *in vitro*.

**Table 5:** Pharmacokinetic and Physiological Properties of 25 selected hits.

**S#**	**Pharmacokinetics**			**AMES toxicity**	**Acute Oral Toxicity**	**Carcinogenicity**	**Rat Acute Toxicity [LD_50_, mol/kg]**	**Physicochemical properties**
	**HIA**	**BBB**	**CYP inhibition/substrate**					
Z1	Yes (Low)	No	I:CYP3A4,1A2,2C9,2C19 NI:CYP2D6 S:CYP3A4 NS:CYP2C9, 2D6	None	None	None	2.2991	F:C16H15ClN4O2S2,MW: 394.90 g/mol, HA: 25, AHA: 15, Fraction Csp3: 0.25, RB: 7, HBA: 5, HBD: 1, MR: 102.26, TPSA: 130.54 Å^2^
Z2	Yes (High)	No	I: CYP 2C9, 2C19 NI: CYP1A2, 2D6, 3A4, S: CYP3A4 NS: CYP 2C9, 2D6	None	None	None	2.6314	Formula: C21H25N5O4S, MW: 443.52 g/mol, HA: 31, AHA:15, Fraction Csp3: 0.38, RB: 7, HBA: 7, HBD: 1, MR:120.64, TPSA: 114.28 Å^2^
Z3	Yes (High)	No	I: CYP 2C9, 2C19, 3A4, NI: CYP 1A2, 2D6, S: CYP 3A4 NS: CYP 2C9, 2D6	None	None	None	2.5609	F: C22H25FN2O5S2, MW: 480.57 g/mol, HA: 32, AHA: 12, Fraction Csp3: 0.41, RB: 7, HBA: 7, HBD: 1, MR: 122.67, TPSA: 118.62 Å^2^
Z4	Yes (High)	No	I: CYP 1A2, 2C9, 2C19, NI: CYP 2D6, 3A4, S: CYP 3A4 NS: CYP 2C9, 2D6	None	None	None	2.4994	F: C22H20N4O3S, MW: 420.48 g/mol, HA: 30, AHA:21, Fraction Csp3: 0.23, RB: 7, HBA: 7, HBD:0, MR: 115.85, TPSA: 115.33 Å^2^
Z5	Yes (Low)	No	I: CYP 2C9, 2C19, 3A4, NI: CYP1A2, 2D6, NS: CYP 3A4, 2C9, 2D6.	None	None	None	2.6457	F:C16H15BrN4O3S3, MW:487.41 g/mol, HA:27, AHA:15, Fraction Csp3: 0.25, RB:7, HBA: 6, HBD: 1, MR:111.55, TPSA: 163.83 Å^2^
Z6	Yes (High)	No	I: CYP2C9, 2C19 NI: CYP1A2, 2D6, 3A4, NS: CYP 3A4, 2C9,2D6	None	None	None	2.4414	F: C20H16NO6S, MW:398.41 g/mol, HA:28, AHA:12, Fraction Csp3: 0.20, RB:7, HBA:6, HBD: 0, MR: 103.83, TPSA: 129.11 Å^2^
Z7	Yes (High)	No	I: CYP3A4, 1A2, 2C9, 2C19, NI: CYP2D6, S: CYP 3A4, NS: CYP2C9, 2D6	None	None	None	2.3806	F: C21H21NO5S, MW:399.46 g/mol, HA:28, AHA:17, Fraction Csp3: 0.24, RB:7, HBA:6, HBD: 0, MR: 108.36, TPSA: 95.12 Å^2^
Z8	Yes (High)	No	I: CYP2C9, 2C19, 3A4, NI: CYP1A2, 2D6, S:CYP3A4, NS: CYP2C9, 2D6	None	None	None	2.6288	F: C22H24N2O4S2, MW:444.57 g/mol, HA:30, AHA:17, Fraction Csp3: 0.32, RB:5, HBA:6, HBD:0, MR: 122.39, TPSA: 105.35 Å^2^
Z9	Yes (High)	No	I: CYP3A4, 1A2, 2C9, 2C19, NI: CYP2D6, S: CYP3A4, NS: CYP 2C9, 2D6	None	None	None	2.3057	F: C22H23N3O4, MW: 393.44 g/mol, HA: 29, AHA:17, Fraction Csp3: 0.32, RB: 8, HBA:6, HBD:1, MR: 107.88, TPSA: 86.48 Å^2^
Z10	Yes (High)	No	I: CYP2C19, NI: CYP 3A4, 1A2, 2C9, 2D6, S: CYP 3A4, NS: CYP 2C9, 2D6	None	None	None	2.2441	F:C21H16BrF3N2O4,MW:497.26 g/mol, HA:31, AHA:12, Fraction Csp3: 0.29, RB:6, HBA: 7, HBD:1, MR: 111.84, TPSA: 75.71 Å^2^
Z11	Yes (Low)	No	I: CYP2C9, 2C19 NI: CYP 1A2, 2D6, 3A4, NS: CYP 2C9, 2D6, 3A4	None	None	None	2.1773	F: C21H14N4O3S2, MW:434.49 g/mol, HA: 30, AHA:20, Fraction Csp3: 0.10, RB:5, HBA:5, HBD:1, MR: 119.98, TPSA: 148.74 Å^2^
Z12	Yes (Low)	No	I: CYP1A2, 2C9, 2C19, NI: CYP2D6, 3A4, S: CYP3A4, NS: CYP2C9, 2D6	None	None	None	2.4932	F:C16H12F3N5O3S, MW:411.36 g/mol, HA: 28, AHA: 16, Fraction Csp3: 0.25, RB: 6, HBA: 11, HBD:0, MR:92.74, TPSA: 133.68 Å^2^
Z13	Yes (Low)	No	I: CYP 2C9, 3A4, NI: CYP 1A2, 2D6, 2C19, S: CYP3A4, NS: CYP2C9, 2D6	None	None	None	2.5971	Formula:C22H22N4O4S2, MW: 470.56 g/mol, HA: 32, AHA: 19, Fraction Csp3: 0.23, RB: 7, HBA: 6, HBD: 2, MR: 127.34, TPSA: 148.85 Å^2^
Z14	Yes (Low)	No	I: CYP3A4, 1A2, 2C9, 2C19, NI: CYP 2D6, S: CYP 3A4, NS: CYP 2C9, 2D6	None	None	None	2.2576	F: C15H13BrN4O2S2, MW: 425.32 g/mol, HA: 24, AHA:15, Fraction Csp3:0.20, RB: 6, HBA:5, HBD:1, MR: 100.14, TPSA:130.54 Å^2^
Z15	Yes (High)	No	I: CYP 1A2, 2C19 NI: CYP 1A2, 2C9, 2D6, 3A4, NS: CYP 2C9, 2D6, 3A4	None	None	None	2.5318	F: C19H17FN4O2, MW: 352.36 g/mol, HA: 26, AHA: 17, Fraction Csp3: 0.21, RB: 6, HBA: 5, HBD: 2, MR: 95.69, TPSA:80.05 Å^2^
Z16	Yes (High)	No	I: CYP 3A4, 1A2, 2C9, 2C19, NI: CYP 2D6, NS: CYP2C9, 2D6, 3A4	None	None	None	2.3583	F:C24H22FN3O4S, MW: 467.51 g/mol, HA: 33, AHA: 18, Fraction Csp3: 0.17, RB:8, HBA:6, HBD: 2, MR: 126.66, TPSA: 103.96 Å^2^
Z17	Yes (High)	No	I: CYP1A2, 2C9, 2C19 NI: CYP2D6, 3A4 S: CYP3A4 NS: CYP2C9, 2D6	None	None	None	2.5253	F:C22H17ClN4O3, MW: 420.85 g/mol, HA: 30, AHA: 21, Fraction Csp3: 0.14, RB: 4, HBA: 5, HBD: 1, MR:113.93, TPSA:78.27 Å^2^
Z18	Yes (High)	No	I: CYP2C9, NI: CYP 1A2, 2C19, 2D6, 3A4, S: CYP 3A4, NS: CYP 2C9, 2D6	None	None	None	2.4841	F: C22H29N3O5S2, MW:479.61 g/mol, HA: 32, AHA: 12, Fraction Csp3: 0.41, RB: 8, HBA: 6, HBD: 1, MR: 130.40, TPSA:120.62 Å^2^
Z19	Yes (High)	No	I: CYP450 1A2, 2C19, 3A4, NI: 2C9, 2D6, NS: CYP2C9, 2D6, 3A4	None	None	None	2.4081	F: C21H20N4O4, MW: 392.41 g/mol, HA: 29, AHA: 17, Fraction Csp3:0.29, RB: 6, HBA: 6, HBD: 2, MR: 106.60, TPSA:98.51 Å^2^
Z20	Yes (High)	No	NI: CYP2C9, 1A2, 2C19, 2D6, 3A4, NS: CYP2C9, 2D6, 3A4	None	None	None	2.8120	F: C21H19N2O6, MW: 395.39 g/mol, HA: 29, AHA: 16, Fraction Csp3: 0.19, RB: 6, HBA: 7, HBD: 1, MR: 107.47, TPSA:113.71 Å^2^
Z21	Yes (High)	No	I: CYP2C9, 2C19 NI: CYP1A2, 2D6, 3A4 NS: CYP2C9, 2D6, 3A4	None	None	None	2.4276	F:C24H26N2O5, MW: 422.47 g/mol, HA: 31, AHA: 16, Fraction Csp3: 0.38, RB:7, HBA: 6, HBD: 0, MR: 118.49, TPSA:79.65 Å^2^
Z22	Yes (Low)	No	I: CYP 2C9, 2C19, NI: CYP 1A2, 2D6, 3A4 NS: CYP 2C9, 2D6, 3A4.	None	None	None	2.2602	F:C22H18FN3O2S2,MW: 439.53 g/mol, HA: 30, AHA:21,Fraction Csp3:0.14, RB:5, HBA:4, HBD:1, MR:119.82, TPSA:131.52 Å^2^
Z23	Yes (High)	No	I: CYP 1A2, 2C19, 2C9 NI: 2C9, 2D6, 3A4, NS: CYP450 2C9, 2D6, 3A4	None	None	None	2.3985	F:C19H17FN2O3S2,MW: 404.48 g/mol, HA: 27, AHA: 15, Fraction Csp3:0.32, RB: 4, HBA: 5, HBD: 0, MR:105.62, TPSA:114.73 Å^2^
Z24	Yes (High)	No	I: CYP450 2C9, 2C19, 3A4, NI: CYP 1A2, 2D6 S: CYP450 3A4, NS: CYP450 2C9, 2D6	None	None	None	2.3289	F: C25H22N4O4, MW: 442.47 g/mol, HA: 33, AHA: 24, Fraction Csp3:0.12, RB: 8, HBA: 7, HBD:1, MR:124.39, TPSA:95.46 Å^2^
Z25	Yes (High)	No	I: CYP 2C9, 2C19, NI: CYP 1A2, 2D6, 3A4, NS: CYP 2C9, 2D6, 3A4	None	None	None	2.4549	F: C24H17FN4O3, MW: 428.42 g/mol, HA: 32, AHA: 24, Fraction Csp3:0.08, RB: 5, HBA: 7, HBD:1, MR: 115.74, TPSA:86.23 Å^2^

*I, Inhibitor; NI, Non-inhibitor; S, Substrate; NS, non-substrate; F, Formula Molecular Weight; HA, Number of Heavy Atoms; AHA, Number of aromatic heavy atoms, Fraction Csp3; RB, Number of rotatable bonds; HBA, Number of H-bond acceptors; HBD, Number of H-bond donors; MR, Molar Refractivity; TPSA, Topological polar surface area*.

## Discussion

Computational methods are extensively used in medicinal and pharmaceutical chemistry researches to foster drug discovery process. *In silico* drug design has given several novel molecules that are in clinical trials. Hence considering the importance of computational drug discovery methods, this study was conducted to discover potential Dengue Virus non-structural protein (DENV NS-3) Helicase inhibitors. DENV NS-3 Helicase is an important drug target to design novel antiviral compounds for the treatment of Dengue.

**Figure 3:**
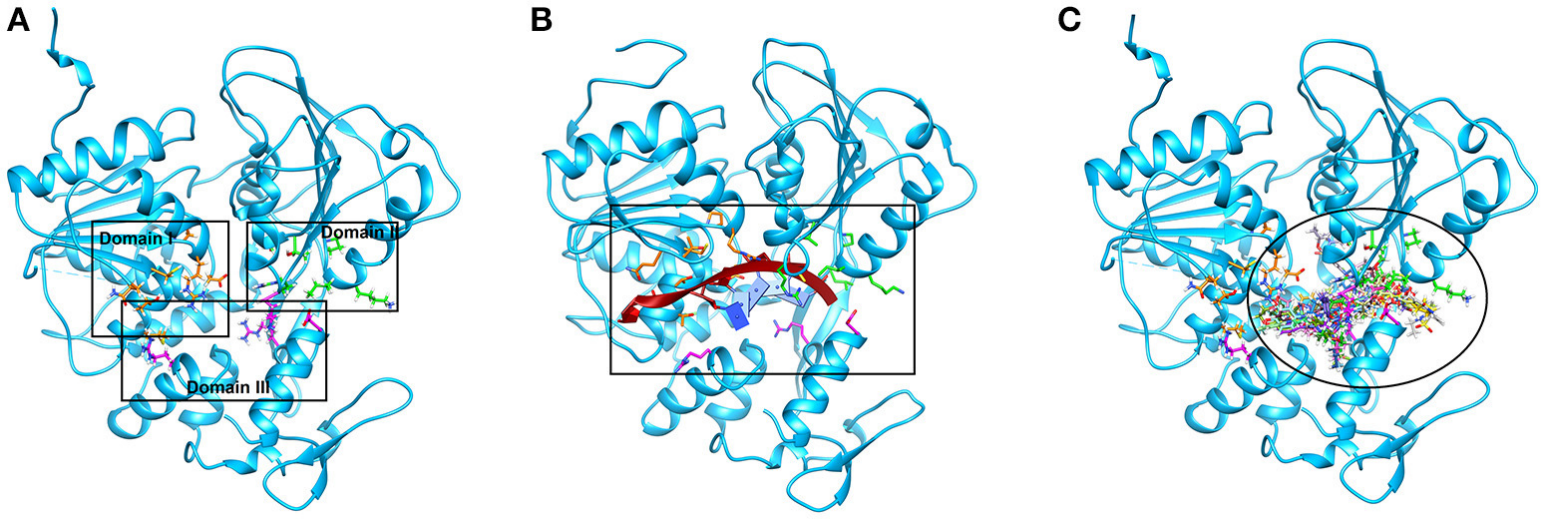
The single stranded RNA binding site is shown **(A,B)**. The residues of domain I, II, and III are shown in orange, green, and magenta color respectively. The docked view of 25 selected hits shows the compounds are fitted between domain II and III **(C)**.

Successful docking studies have been performed in the recent years (Zaheer-ul-Haq et al., 2010; Halim et al., 2015). Recently several novel immunomodulators were designed using *in silico* approaches when screened against Interleukin-2 (Halim et al., 2013). Knowing the importance of computational drug designing methods, in this research, ligand-based pharmacophore modeling was carried out. The pharmacophore model was validated by screening known inhibitors embedded in compounds library collected from ZINC dataset. Furthermore, 1201474 compounds selected from drug-like category of ZINC database was screened by pharmacophore model which led to identify 694 molecules that were subjected to docking by FRED docking suit. The compounds were scored by eight scoring functions (Shapegauss (SG), Piecewise Linear Potential (PLP), ChemScore, Chemgauss 2 (CG2), and Chemgauss 3 (CG3), OeChemScore, ScreenSscore, and Zapbind) to evaluate the performance of selected scoring functions. Shapegauss is a shape based scoring function that select the best pose based on its shape complementarity with active site, but lack estimation of protein-ligand interactions. PLP estimates shape and protein-ligand interactions specifically hydrogen bonding. Chemscore estimates lipophilic interactions, H-bonding, metal/ligand interactions as well as any rotatable bonds in a pose and clash between protein-ligand. CG2 and CG3 are Gaussian scoring functions that calculate shape complementarity, however CG3 also calculate H-bonding between protein and ligand, between ligand and solvent and metallic interactions. OEChemscore is a variant of chemscore, but it is unable to calculate entropy penalty upon complex formation. Screenscore is a hybrid of PLP and FlexX scoring functions; it calculates interactions between polar and non-polar atoms. Zapbind is the most computationally expensive scoring function among all the ones integrated in FRED. It sum up surface area contact term (calculated by Gaussian-based method) and an electrostatic interaction term calculated using the Poisson–Boltzmann (PB) solvent approximation which is calculated by ZAP. Prior to virtual screening experiments the comparison of available scoring functions must be conducted to ensure the suitable scoring function for the target of interest (Zaheer-ul-Haq et al., 2010). For this purpose, retrospective docking analysis gives good idea of which scoring function is best for the protein under observation. Among eight scoring functions, CG2 and SG showed excellent results and predicted all the 16 actives as top ranked inhibitors. Hence the CG2 and SG top ranked ZINC compounds were predicted as efficient inhibitors of DENV NS-3 Helicase. Interaction analysis revealed that 25 compounds significantly showed good interaction with the NS3 Helicase active site. The compounds showed strong hydrogen bonding interactions with the target protein. A model of nucleic acid binding site of NS3 helicase was generated by (Xu et al., 2005). The model revealed interaction between the NS3 nucleotide binding-site with a deoxyuridylate octamer oligonucleotide (single stranded RNA). The oligonucleotide interacted with residues from motifs Ia, IV, and V, and residues Arg-225 (motif Ia), Lys-366 (motif IV), Arg-387, Lys-388, and Arg-538 and Arg-599 from domain III interacted with the phosphodiester backbone. These residues (Arg-225, Lys-366, Arg-387, Lys-388, Arg-538, and Arg-599) binds with the single stranded RNA. However, single stranded RNA binding site was elucidated by Luo et al. (2008). The ssRNA binds with domain I, II and III. The residues Pro223, Arg225, Asp290, Gln243, Thr244, Cys261, Thr264, Thr267 from domain I, Pro363, Ile365, Lys366, Arg387, Thr408, Asp409, Leu429 from domain II, Arg538, and Arg599 and Asp603 from domain III makes a tunnel to accommodate ssRNA. The crystallographic structures of DENV NS-3 Helicase in complex with ssRNA (PDB ID: 2JLU), with ADP (PDB ID: 2JLS), with ligand ANP (phosphoaminophosphonic acid adenylate ester, PDB ID: 2JLR) and with ANP and ssRNA (PDB ID: 2JLV) showed that NTPase/ATPase and helicase active site are distinct (Luo et al., 2008). Our docking results showed that most of the compounds interacted with Arg599, Lys388, and Arg387. Hence the compounds are accommodated in a groove between domain II and III. The interactions are depicted in Figure 3.

Computational tools also aid in prediction of ADMET properties of compounds. ADMET prediction is essential to remove compounds that show toxicity in biological system. Thus, prior to *in vivo* trial ADMET properties of selected compounds must be calculated to remove any toxic compound. These assessments further increase the efficacy of drug candidate and reduce the chance of its failure in *in vivo* trials. Thus, ADMET profiling was conducted *via* admetSAR and all the compounds revealed good pharmacokinetic properties. These results suggest that these compounds can be considered as potent inhibitors of DENV NS3 Helicase by hindering its active site.

## Conclusion

Treatment of Dengue is one of the main public concerns nowadays therefore novel inhibitors need to be urgently designed to cure this disease. DENV NS-3 Helicase is a potential drug target. We employed computational modeling techniques including ligand based pharmacophore modeling and structure based virtual screening to identify novel and potential DENV NS-3 Helicase inhibitors from ZINC database. The *in silico* results demonstrated 25 hits compatible with active site of NS-3 Helicase and are predicted to block its activity *in silico*. The current computational results will be validated in wet lab by both *in vitro* and *in vivo* testing.

## Author contributions

SH outline the research strategy and idea. SK carried out the literature search, and performed computational experiments. SH drafted and revised the manuscript. All authors read and approved the final manuscript.

### Conflict of interest statement

The authors declare that the research was conducted in the absence of any commercial or financial relationships that could be construed as a potential conflict of interest. The reviewer, TM, and handling Editor declared their shared affiliation.
